# Hydrogels in Ophthalmology: Novel Strategies for Overcoming Therapeutic Challenges

**DOI:** 10.3390/ma17010086

**Published:** 2023-12-23

**Authors:** Kevin Y. Wu, Dania Akbar, Michel Giunta, Ananda Kalevar, Simon D. Tran

**Affiliations:** 1 Department of Surgery, Division of Ophthalmology, University of Sherbrooke, Sherbrooke, QC J1G 2E8, Canada; yang.wu@usherbrooke.ca (K.Y.W.);; 2 Department of Human Biology, University of Toronto, Toronto, ON M5S 1A1, Canada; 3 Faculty of Dental Medicine and Oral Health Sciences, McGill University, Montreal, QC H3A 1G1, Canada

**Keywords:** hydrogels, material science, polymers, ophthalmology, intravitreous injection, suprachoroidal injection, cell-based therapy, intraocular lens, biocompatibility, drug delivery

## Abstract

The human eye’s intricate anatomical and physiological design necessitates tailored approaches for managing ocular diseases. Recent advancements in ophthalmology underscore the potential of hydrogels as a versatile therapeutic tool, owing to their biocompatibility, adaptability, and customizability. This review offers an exploration of hydrogel applications in ophthalmology over the past five years. Emphasis is placed on their role in optimized drug delivery for the posterior segment and advancements in intraocular lens technology. Hydrogels demonstrate the capacity for targeted, controlled, and sustained drug release in the posterior segment of the eye, potentially minimizing invasive interventions and enhancing patient outcomes. Furthermore, in intraocular lens domains, hydrogels showcase potential in post-operative drug delivery, disease sensing, and improved biocompatibility. However, while their promise is immense, most hydrogel-based studies remain preclinical, necessitating rigorous clinical evaluations. Patient-specific factors, potential complications, and the current nascent stage of research should inform their clinical application. In essence, the incorporation of hydrogels into ocular therapeutics represents a seminal convergence of material science and medicine, heralding advancements in patient-centric care within ophthalmology.

## 1. Introduction

The intricate anatomical and physiological design of the human eye introduces distinct challenges, both pharmacologically and surgically, when managing various ocular pathologies [1]. Over recent years, there has been a concerted effort in the ophthalmological community to improve the delivery of therapeutic agents and enhance surgical interventions. One avenue that has gained attention in this pursuit is the use of hydrogels.

Hydrogels offer a promising approach in ophthalmology, primarily due to their biocompatibility, adaptability, and potential for customization. These attributes render them valuable in a range of applications, from facilitating drug delivery in the posterior segment to optimizing postoperative outcomes in cataract surgery through intraocular lens technology [2].

**Figure 1:**
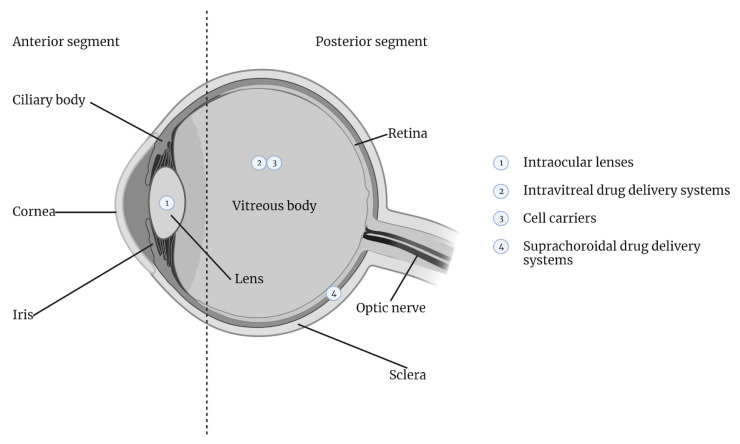
Current state of hydrogel applications in the field of ophthalmology.

In this review, we present the findings from a comprehensive literature review focused on preclinical and clinical studies published within the last five years. This review aims to offer a comprehensive exploration of the current state of hydrogel applications in the field of ophthalmology. Beginning with an analysis of the anatomical and functional nuances of the posterior segment, which underscore the significance of targeted and sustained drug delivery, we traverse through the various hydrogel-based approaches to intravitreal (IVT), suprachoroidal, and cell-based therapies (Figure 1). Furthermore, an examination is devoted to the exciting realm of hydrogel-utilized intraocular lens technology, heralding potential advancements in postoperative care, drug delivery, and even disease diagnostics. Through this analysis, we aim to shed light on the current advancements and the prospects of these “smart molecules” in enhancing patient outcomes and addressing the multifaceted challenges inherent to ocular care. In other words, our review article provides a nuanced examination of hydrogel technologies in ophthalmology, incorporating recent therapeutic and surgical applications. It offers an update to the field by including emerging administration techniques like suprachoroidal injections, which have received limited attention in prior reviews. This article, enriched with clinical insights from ophthalmic surgeons, carefully assesses the role of hydrogels, suggesting a measured evolution of these materials from experimental to clinical settings.

## 2. Overview of Hydrogels

Over the past 60 years, the biological use of hydrogels in various medical applications has significantly increased [3,4]. Today, they are used almost everywhere, such as in the fields of agriculture, environmental engineering, soft robotics, and the food industry, and continue to be explored for new applications and improved for existing ones [3]. Before expanding on the current diverse applications of hydrogel-based drug delivery systems (DDS) in ocular therapy, it is essential to understand what hydrogels are, why they are good delivery systems, how they can be classified, and how they are synthesized.

### 2.1. Definition and Properties

Hydrogels are three-dimensional (3D) polymer chains that swell in water and can thus hold large amounts of water while maintaining their structural integrity. Their function is mainly achievable due to their unique network that encompasses crosslinked polymer chains by the chemical, physical, or composite cross-linking of individual chains [2,3]. As such, hydrogels serve as a self-healing biomaterial with extensive applications [5]. This is possible due to the presence of polar hydrophilic moieties within the polymer network which can bind the water molecules [2]. For a material to constitute a hydrogel, it must have at least 10% of the total weight (or volume) of water [6]. The swelling nature of the hydrogel can be controlled by the strength and number of attached hydrophilic groups, the swelling media, and the strength of the cross-linked material [2,7,8]. Due to the high water content, hydrogels have flexibility and softness similar to living tissue, thus making them a suitable material for in vivo use as a biomimetic. Hydrogel swelling also provides protection to the loaded cargo from degradation or premature dispersal in the body [7]. They have ideal properties for biomedical applications including biodegradability, biocompatibility, and hydrophilicity, which is essential especially when considering applications in highly aqueous organs such as the eye [3,5,7,9,10]. Perhaps the greatest advantage of hydrogels when compared to other nanomaterials is that they can be thermoresponsive—injectable in vivo as a liquid which then undergoes a reversible gelation at body temperature, allowing for the flexibility required to penetrate into deeper regions [11,12,13]. Many, if not all, components of the human body are also viscoelastic, another property which is observed in hydrogels [14,15]. The combination of the viscous behavior of fluids with the elastic behavior of solids allows the gels to both temporarily flow and deform under some force before returning back to their original state [14,15]. This supports easier penetration into the organs and inner segments whilst combatting the various forces that the body faces and preserves the hydrogel’s properties and function [14,15]. Hydrogels are a suitable DDS, as they can be modified to meet the mechanical requirements of the region of interest. 3D hydrogels have the capacity to respond to specific stimuli and to effectively deliver drugs to the posterior segment of the eye, a persisting challenge in ophthalmology [8]. They further allow for an increased loading capacity, thus increasing drug concentrations, and have a predictable release profile which can be studied through computational modeling, allowing for accurate ex vivo modulation of the ideal hydrogel properties for sustained release [16].

### 2.2. Classification of Hydrogels

**Figure 2:**
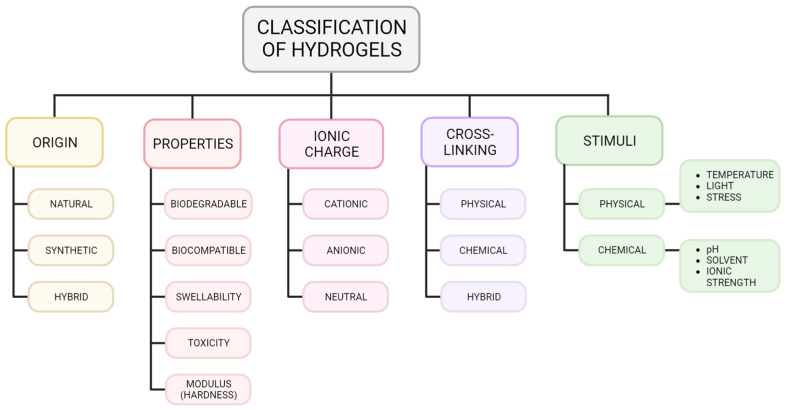
Classification of Hydrogels.

Hydrogels can be classified based on several properties (Figure 2), which will be discussed in detail below. Classification can depend on the properties of the hydrogel such as biocompatibility, swellability, and stimulus sensitivity, which depend on how the hydrogel is synthesized (Figure 2) [3,10,17,18]. They can be categorized based on their ionic charge, where the charge of the hydrogel network depends on the individual polymer charges and may be a net positive, negative, or neutral [10,19]. The cross-linking method and the type of cross-linking may also differ—either physical or chemical—and modulate the strength and stability of the polymer network [16]. The hydrogel composition is another important component, broken down into either an interpenetrating network (IPN), a co-polymer, or a homopolymer [3,20]. IPN hydrogels combine two or more polymeric units cross-linked together, which is advantageous due to their viscoelastic properties and easy swelling [20]. Homopolymer hydrogels contain one monomer species cross-linked into a 3D network, whereas co-polymers contain multiple monomer species which are cross-linked together [20]. However, both contain only one type of polymer chain compared to IPNs. Lastly, the response of the hydrogel to chemical stimuli (e.g., pH, ionic strength, solvent composition) or physical stimuli (e.g., temperature, light, pressure, mechanical stress) serves as a classifier of hydrogels and is important when considering how the hydrogel will behave in vivo [10,18].

Polymers used for hydrogels range from natural, semisynthetic, and synthetic materials which can be used either alone or as starting materials onto which modifications are added for enhanced function (Table 1). Polymers of natural origin, such as collagen, chitosan, gelatin, alginate, and hyaluronic acid (HA), have the advantage of being biodegradable and non-toxic and are obtained from renewable sources [8,9,10,21]. Natural polymers can easily interact with biological components such as the extracellular matrix (ECM), cells, and protein components. This is desirable for sustained release, localized delivery, and cell therapy, where the penetration of encapsulated cells and their secretions into host tissue is vital for permanent efficacy [22]. They are also simpler to synthesize and capable of absorbing large quantities of toxic agents. Natural hydrogels typically demonstrate a lower mechanical resistance, attributed to their lower-density and low-friction polymer chains [23]. However, it is also essential to consider the nature of their elasticity. Hydrogels with flexible, less stiff polymer chains exhibit entropy elasticity where mechanical properties are influenced by the polymer chains’ tendency to return to a state of maximum disorder by returning to their original size [24]. Conversely, hydrogels with stiffer polymer chains may display energy elasticity, where resistance to deformation is dictated by the energy required to alter the bonds within the chain [25]. This distinction in the type of elasticity plays a crucial role in the mechanical properties of hydrogels. Modifications with synthetic materials are often employed to enhance these properties and mitigate the inherent weaknesses of natural polymers [23].

Conversely, synthetic hydrogels such as poly(ethylene glycol) (PEG), poly(acrylic acid) (PAA), and N–isopropyl acrylamide (NIPAAm) allow for greater water absorption, an expanded library of materials, and more control of the composition and mechanical properties (Table 1) [9,20,21]. Additionally, synthetic hydrogels have a longer life and higher resistance, allowing for a longer sustained delivery [21]. However, while many are non-toxic and biocompatible to some extent, synthetic hydrogels have a lower cytocompatibility and greater foreign body response compared to natural hydrogels [2]. In some applications, the base materials alone, such as PEG, also do not provide a suitable environment for cell adhesion, and thus, modifications are required, adding to the complexity and risk of the material [26].

In other instances, a blended hydrogel may be most effective, combining the biocompatibility of natural hydrogels with the increased mechanical strength and stability of synthetic materials to maximize the efficiency of the hydrogel (Table 1) [21]. Such hybrid hydrogels include polysaccharides and synthetic polymers such as PEG conjugated with ECM components like fibronectin, gelatin, albumin, elastin, or collagen. These semi-synthetic scaffolds provide greater structural support for cells, allowing for cell regeneration and growth, as well as a temporal and spatial control over cell signaling biomolecules involved in cell regeneration [27]. Furthermore, the degradation rate can be controlled, which can, in return, enable adequate cell repair pathway activations [27]. It is important to note that the choice of ECM components is crucial; they can provide different spectrums of elasticity, immunogenicity, affinity, proteolytic susceptibility, and microenvironment specificity.

### 2.3. Hydrogel Synthesis and Challenges in Ophthalmology

**Figure 3:**
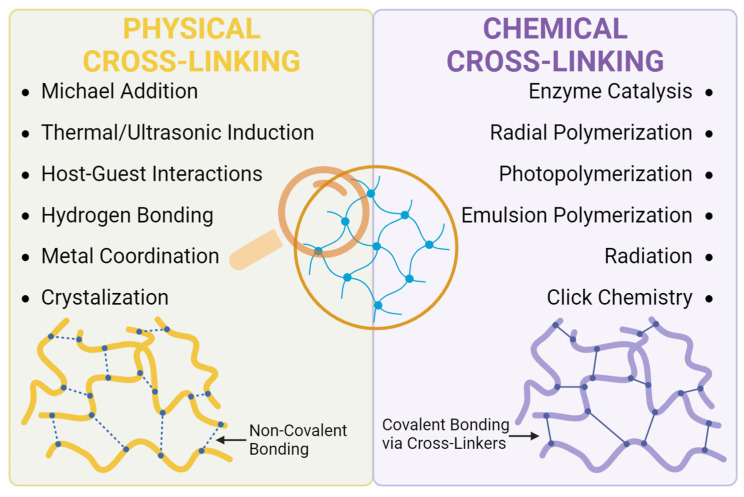
Methods of Chemical and Physical Cross-linking of Hydrogels.

Hydrogels are typically either physically or chemically cross-linked during synthesis, depending on the nature of the bonds connecting the polymeric molecules together (Figure 3) [16,20]. It is these bonds that provide hydrogels with stability and structure. The type and number of bonds allow for tunability of the hydrogel properties [20]. Physical cross-linking is achieved when the polymer chains interact through ionic bonds, Van der Waals forces, hydrogen bonding, hydrophobic interactions, or complexation [10,16]. Many techniques have been used in order to achieve the physical cross-linking of hydrogel polymers, such as Michael addition, thermal induction, ultrasonic induction, crystallization, H-bonding, metal coordination, and host–guest interaction [28]. These hydrogels are reversible but generally less stable, as the bonds are susceptible to breaking if there are changes in the pH, temperature, ionic strength, or the presence of mechanical stress [8,9]. One of the main advantages of physically crosslinked hydrogels is their safety due to the lack of cytotoxicity induced by chemicals.

Chemically cross-linked hydrogels are considered “permanent,” as the interactions between the polymer chains are covalent bonds [16]. These hydrogels can be synthesized in several ways, most commonly through the radial polymerization of monomers, radiation, enzyme catalysis, or the addition of cross-linking agents (e.g., diacrylate or glutaraldehyde) [10,16,21]. Common reactions for conducting chemical cross-linking for hydrogels include Schiff base formation, Diels–Alder reactions, click chemistry, or Michael-type addition reactions among others [21]. Given the more permanent bondage, chemically cross-linked hydrogels exhibit a tougher structure and are less prone to degradation [16,22]. However, this may be a disadvantage where rapid degradation is required, such as for cell-therapies [16,22]. Chemical cross-linking has rapid in situ gelation following injection, which influences the encapsulation efficacy and release profile of loaded cargo [22]. The cross-linking material is another important consideration, as it should be non-toxic and not interfere with the desired function of the hydrogel [16]. For a more detailed discussion on the cross-linking interactions of hydrogels, several recent articles are suggested [7,9,10,16].

In ophthalmic applications, several factors must be taken into consideration when designing a hydrogel. For one, due to the unique function of the eye, most hydrogels should be transparent or semi-transparent to preserve vision, particularly as many of the diseases of interest already have resultant vision loss [13,29]. In cases of vitreous substances, contact lenses, and intraocular lenses, transparency is a key component that cannot be compromised. Hydrogels for tissue engineering and cell-based applications should have a high capacity for rapid self-healing, as well as a safe degradation, which would allow encapsulated tissue to engraft to the host eye [30]. Another consideration is that the hydrogel should have biomimetic properties suitable for the ocular region of delivery [8]. It should also have an optimal gelation, which allows for facile injection through small-gauge needles. The gel needs to be soft enough to not disrupt native structures whilst preserving the mechanical integrity needed for long-term therapeutic effect [8,13,18]. Several hydrogels are also restricted in their biomedical uses at the moment due to limited degradability and biocompatibility, and those that do meet the criteria are rather understudied to date [9]. Focus needs to be placed on developing successful long-term hydrogels which maintain their mechanical and physical properties and overcome the challenges associated with existing therapies. Nonetheless, significant advancements are underway for optimizing hydrogel-based delivery in several ophthalmic applications, which will be discussed below.

## 3. Ocular Anatomy and Physiology

### 3.1. Anatomical and Functional Complexity of the Posterior Segment of the Eye

**Figure 4:**
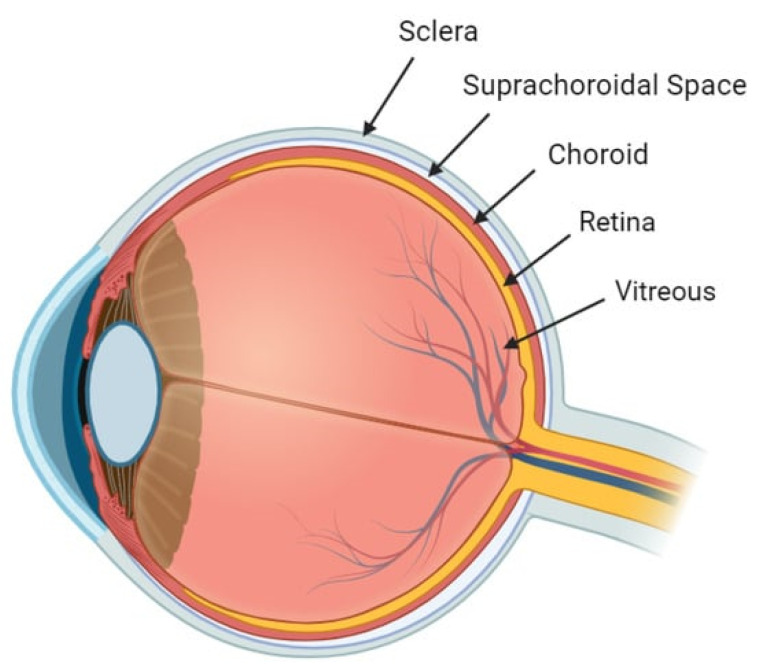
Anatomy of the Posterior Segment.

The anatomical and functional nuances of the eye’s posterior segment are central to this review, which delves into the applications of hydrogel-based DDS for ocular conditions (Figure 4). A nuanced understanding of ocular anatomy is essential for optimizing these “smart molecules” as advanced DDS. This foundational knowledge also informs the selection of appropriate administrative routes, illuminating both the potential and constraints of hydrogel DDS.

**Figure 5:**
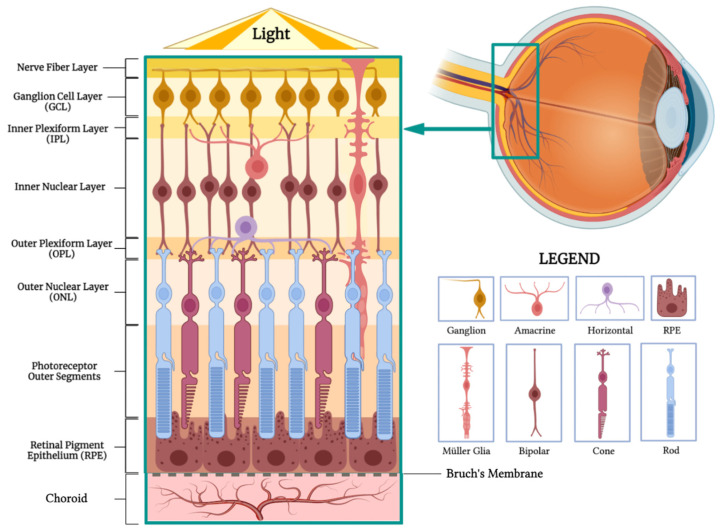
Retinal Anatomy. The illustration highlights the different layers of the retina and its main cell types. (BioRender, https://app.biorender.com/, accessed on 17 July 2023.)

The retina, originating from the optic cup’s inner and outer layers, is a multilayered structure integral to visual processing (Figure 5). In a proximal to distal arrangement, it consists of 10 specialized layers [31]:

Nerve Fiber Layer: Houses axons of retinal ganglion cells, converging to form the optic nerve.

Ganglion Cell Layer: Contains cell bodies of retinal ganglion cells that relay visual data to the brain.

- Inner Plexiform Layer: Facilitates synaptic interactions between bipolar and ganglion cells, crucial for signal integration.
- Inner Nuclear Layer: Hosts the nuclei of bipolar, horizontal, and amacrine cells, which are essential for visual signal processing.
- Outer Plexiform Layer: Features synapses between photoreceptors and bipolar or horizontal cells, vital for initial signal transduction.
- Outer Nuclear Layer: Comprises rod and cone photoreceptor cell bodies, responsible for capturing and translating light into neural impulses.
- Rod and Cone Segments: The functional parts of photoreceptor cells, where light absorption and phototransduction occur.
- Retinal Pigment Epithelium (RPE): This outermost layer absorbs stray light and nourishes the photoreceptors, among other functions.

Horizontal cells interact synaptically with rods and cones, while bipolar cells connect vertically, synapsing with either rods or cones. These bipolar cells eventually synapse with ganglion and amacrine cells in the inner plexiform layer. The collected axons from ganglion cells make up the optic nerve, comprising over one million nerve fibers [32].

This intricate arrangement underscores the retina’s role as a complex sensory organ, with each layer contributing uniquely to the visual pathway.

Directly abutting the retina is the choroid, a vascular layer essential for nourishing the retina and especially its metabolically active outer portions, including the photoreceptors and the RPE. Supplied by the posterior ciliary arteries, the blood traverses through the Haller and Sattler layers before arriving at the choriocapillaris, where arterial pressure significantly declines. The choroid’s thickness is variable, measuring an average of 287 μm subfoveally in a typical 50-year-old subject, but it can differ with age and disease [33]. Functionally, it accounts for nearly 90% of the oxygen uptake by the outer retina, efficiently handling nutrient delivery and waste removal [32].

Adjacent to the choroid is the Suprachoroidal Space (SCS), a potential area maintained in close contact with the sclera primarily due to intraocular pressure (IOP) and specific attaching fibers [34,35]. Its dimensions can be manipulated pharmacologically, as demonstrated by triamcinolone acetonide injections that temporarily expanded the SCS from a mean width of 9.9 to 75.1 μm [36]. Anatomically, the SCS is delineated by specific boundaries, extending anteriorly to the scleral spur and posteriorly near the optic nerve and short posterior ciliary arteries [37,38,39,40]. This anatomical insight is pivotal for interventions like Suprachoroidal (SC) injections. Unlike the subretinal space, the SCS is not immunologically privileged due to its external location in relation to the blood–retinal barrier, which comprises a non-fenestrated vascular endothelium and tight junctions in the RPE [37,38,39,40].

### 3.2. Static and Dynamic Barriers of the Eye

#### 3.2.1. Corneal Barrier

**Figure 6:**
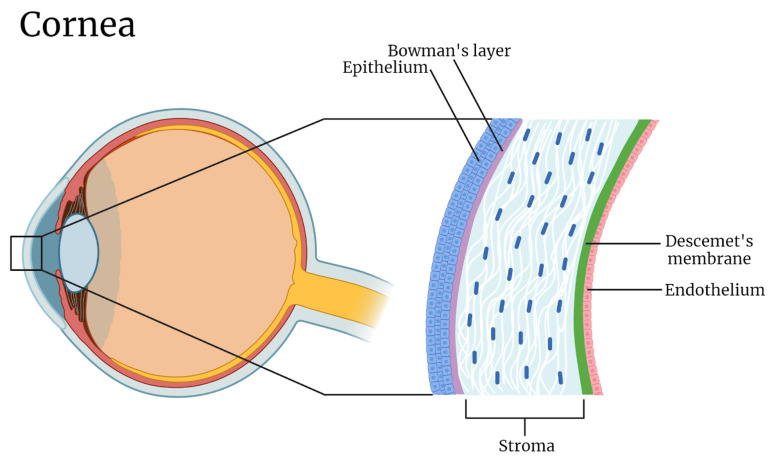
Corneal Barrier.

Serving as the eye’s defensive barrier, the cornea boasts a five-layered architecture, encompassing the epithelium, Bowman’s membrane, stroma, Descemet’s membrane, and endothelium (Figure 6). This sophisticated arrangement plays a pivotal role in mediating the entry of therapeutic agents. The corneal epithelium, with its intricate interplay of tight and gap junctions, is the primary point of interaction for these substances. Supporting this, the stroma and Descemet’s membrane provide foundational stability for the inner endothelial cells, which are equipped with macula adherens crucial for controlled substance transfer [41].

The cornea’s protective attribute is harmonized with its semi-permeable nature, allowing for selective molecular movement. However, at the corneal epithelium’s level, substances with larger dimensions and hydrophilic tendencies encounter barriers, specifically a layer’s zonula occludens, which only permits passage to substances under 2.0 nm in diameter. The stroma, abundant in collagen, is predominantly hydrophilic, which impedes the passage of lipophilic molecules. Additionally, at a physiological pH, the pores’ negative charge introduces an extra barrier for charged molecules through ionic interactions. Simply put, the cornea acts as a barrier, limiting the penetration and bioavailability of topically administered drugs [42].

Although some drugs can navigate into the aqueous humor via the transcorneal route, they often fail to reach the posterior segments of the eye at therapeutic concentrations due to the vitreous barriers [1].

#### 3.2.2. Vitreous Barrier

IVT drug administration stands as a direct conduit to the vitreous and retina. Yet, challenges arise from barriers such as the RPE that may impede larger, positively charged molecules from travelling from vitreous to choroid [42].

#### 3.2.3. Aqueous Humor

The aqueous humor primarily employs two drug elimination pathways: the conventional one, engaging the trabecular meshwork and Schlemm’s canal, and the uveoscleral route, which operates based on a drug’s lipophilicity. The former functions through convective flow, independent of drug-specific attributes [43]. In essence, the turnover of the aqueous humor serves to continually wash out drugs introduced via topical, intracameral, or IVT routes [44].

#### 3.2.4. Blood–Ocular Barrier (BOB)

This encompassing barrier merges two integral components: the blood–aqueous barrier (BAB) and the blood–retinal barrier (BRB). Collectively, they serve as formidable guardians of the ocular system [41]. The BAB is demarcated by the endothelial cells of the ciliary body vessels coupled with the pigmented and nonpigmented epithelium, forming a rigorous defense against incoming molecules from the systemic circulation. In parallel, the BRB acts as a crucial line of defense, restricting drug penetration from the bloodstream into the retina. Comprising both the retinal capillary endothelium and the RPE, the BRB ensures selective substance exchange between the retina and the surrounding vascular system [45].

At its core, the blood–ocular barrier (BOB) functions as a protective boundary, diminishing the penetration and bioavailability of systemically administered drugs within the ocular tissue [45,46].

**Figure 7:**
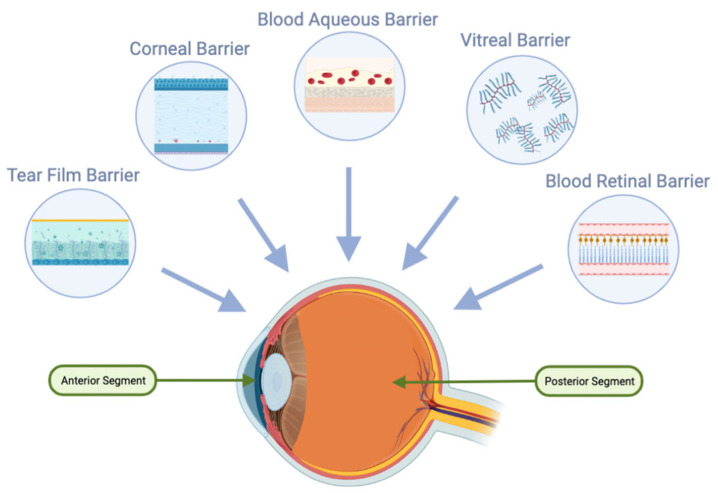
Static and Dynamic Barriers of the Eye. The provided illustration underscores the predominant barriers within the eye, which serve dual purposes: first, to preserve its internal milieu; and second, to pose challenges for the effective delivery of administered drugs. (BioRender, https://app.biorender.com/, accessed on 15 June 2023.)

Figure 7 outlines the aforementioned barriers.

## 4. Intravitreal Drug Delivery Systems

In current ophthalmological practice, IVT injections are frequently employed for the treatment of posterior segment eye diseases (PSED), utilizing therapeutic agents such as anti-vascular endothelial growth factor (anti-VEGF) antibodies and corticosteroids [47]. Specifically, anti-VEGF agents are commonly used to manage retinal diseases characterized by neovascularization, including but not limited to wet age-related macular degeneration (wet AMD), proliferative diabetic retinopathy (PDR), diabetic macular edema (DME), and ischemic retinal vein occlusion (RVO) [6]. Given this landscape, there has been considerable research interest in devising methods that facilitate more localized, controlled, and sustained delivery mechanisms for anti-VEGF agents.

While the current regimen of IVT injections for PSED is clinically effective, it necessitates regular administration to sustain therapeutic benefits [47,48,49]. Such frequent injections are less than ideal for several reasons. Primarily, they contribute to patient discomfort, thereby adversely impacting compliance rates [48]. Additionally, the need for frequent, scheduled visits to healthcare facilities amplifies the economic strain on the healthcare system. Not to be overlooked are the medical risks that accompany these repetitive interventions, including the elevated probability of severe complications like endophthalmitis and retinal detachment [47]. Hydrogels have been demonstrated to potentially alleviate these limitations by potentially extending treatment efficacy. This could, in turn, potentially lead to a reduction in the frequency of required injections [13]. Moreover, the flexibility in the properties of the hydrogel can be tailored to meet individual patient needs [13,50].

Expanding on this, hydrogels serve as an innovative DDS specifically designed for controlled release to the posterior segment of the eye. They provide the added benefits of facilitating concurrent drug delivery and enabling in situ hydrogel formation and degradation. This feature set allows for biocompatible treatment options that bypass many of the constraints commonly associated with IVT implants [13]. The discussion below highlights recent work on both natural and synthetic hydrogels for IVT delivery (Table 2).

### 4.1. Synthetic and Semi-Synthetic Hydrogels for Intravitreal Ocular Drug Delivery

PEG-based hydrogels are among the most studied DDS for the treatment of PSED, mainly due to their versatility and biocompatibility [82]. By modulating polymer concentrations, it is possible to influence drug release pharmacokinetics. Using bevacizumab-loaded tetra-PEG gels, the drug release was shown to be slower at higher PEG concentrations (e.g., 5% and 10%), with a release period spanning over 2 weeks, whereas 1.5% tetra-PEG gels provided a more rapid release pattern [83].

The use of thermo-responsive NIPAAm hydrogels has been explored in recent years for PSEDs such as PDR, wet AMD with choroidal neovascularization (CNV), retinal vein occlusions (RVO), and DME. Thermo-responsive or stimuli-responsive hydrogels, also known as smart hydrogels, are known for their high swelling and gelation capacity [84]. Therefore, they provide an excellent alternative for absorption by hydrophilic or hydrophobic structures and have demonstrated interesting clinical applications in aqueous media. An NIPAAm-PEG-NIPAAm triblock polymer with embedded dexamethasone (DEX) was formulated as a self-healing gel for IVT delivery [51]. DEX release occurred in an aqueous medium for over 430 days at a physiological temperature and pH, and it was suggested using simulations that the administration of 100 mg of hydrogel would be sufficient for a similar release profile in vivo. Furthermore, in vitro experiments have demonstrated the cytocompatibility of thermosensitive hydrogels with the macrophage-like mural cells (RAW 264.7) and human RPE-derived cells [85]. Likewise, Dosmar et al. simulated drug diffusion from four compartment models of the eye, using a computational modeling of vancomycin-loaded polyNIPAAm (PNIPAAm)-PEG diacrylate (PEG-DA) thermos-sensitive hydrogels [52]. Vancomycin was the fastest at reaching peak concentrations following the IVT administration simulation; however, concentrations also dwindled the fastest, whereas topical administration reached higher levels of the drug concentration at a slower rate but was shown to be potentially maintained the longest [52]. The PEG-DA component of the gel was also non-degradable, remaining in vitro for at least 187 days [52]. When administered to Long-Evans rats with *Staphylococcus aureus*-induced acute endophthalmitis, significantly lower infections scores were obtained 24 h post-injection when compared to the control groups [86]. Furthermore, cytotoxicity studies with histopathology analysis yielded promising results; less than 10% of the cell viability was assessed [86]. A PEG-poly(L-lactic acid) (PLLA)-DA modification did, however, demonstrate degradability [52]. Topical delivery is a current standard for posterior segment diseases, and these results provide a possible alternate through IVT given that the drug residence time can be prolonged. The same group has further explored the PEG-PLLA-DA/NIPAAm hydrogel both in vitro and in vivo with encapsulated poly(lactic co-glycolic acid) (PLGA) microspheres for the delivery of aflibercept and ranibizumab [53,54,55,56,57,87]. When tested in a laser-induced rat model of CNV, aflibercept-loaded DDS hada significant reduction in lesion areas for up to 12 weeks, which was maintained up to 6 months with minimal adverse effects on retinal cell function [87]. The DDS is injectable through a 28-gauge needle at room temperature, and as expected, modifying the synthesis temperature, as well as the cross-linker concentration, affected the gel swelling ratio, increased the volume phase transition temperature (VPTT), and facilitated in vitro degradation [54,55,56]. Taking it one step further, they also observed bioactivity with a transient focal foreign-body response for up to 6 months in healthy rhesus macaque monkeys [57]. The next steps would be to test the efficacy in diseased models in non-human primates.

Nonetheless, other composite PEG and PLGA co-polymers are also under exploration for the IVT delivery of anti-VEGF drugs, antivirals, and corticosteroids. Hu et al. designed an mPEG-PLGA-BOX hydrogel with a sol-gel transition at body temperature [58]. The release of bevacizumab—one subtype of an anti-VEGF agent—from the hydrogel was shown to be bioactive for the potential treatment of wet AMD by inhibiting retinal angiogenesis in the eyes of laser-induced photocoagulation Rex rabbits. In a previous study, they also demonstrated an in vitro release profile of approximately 30 days, which is similar to current anti-VEGF delivery systems, and thus, further work should continue to be carried out to evaluate whether this hydrogel can be used as an alternative therapy [88]. Similarly, the crosslinking of 4-arm-PEG-maleimide with HA-bearing furan groups (4APM-HAFU)—a hydrogel with a sol-gel phase transition—was also shown to induce the sustained release capabilities of bioactive bevacizumab [89]. The delivery of the corticosteroid, DEX, was examined by Lopez-Cano et al. in a PLGA-PEG-PLGA triblock co-polymer hydrogel, for which they effectively demonstrated sustained release between 47 to 62 days depending on the hydrogel composition [59]. The gel was well tolerated in the human retinal pigment epithelial (RPE-1) cell line and further improved with the co-delivery of antioxidant agents [59]. DEX release was shown to reduce LPS-induced tumor necrosis factor alpha (TNF-α) production, a major contributor to the inflammatory cascade, thus successfully demonstrating the anti-inflammatory properties of these hydrogel-based DDS in vitro [59]. With a very similar formulation, the substitution of PLGA for poly (β-butyrolactone-co-lactic acid) (PBLA) was proposed for the treatment of cytomegalovirus (CMV) retinitis through the hydrogel-encapsulated delivery of an antiviral drug, ganciclovir (GCV) [60]. Lactic-acid-based hydrogels are thermo-responsive, exhibiting phase changes, especially near body temperatures, making them suitable for easier in vivo drug delivery [60]. The authors observed that more than 85% of GCV could be released from the hydrogel within 96 h, and the gel had a greater half-life in the aqueous humor compared to GCV injection alone [60].

A polyurethane thermogel composed of PEG, poly(propylene glycol) (PPG), and poly(ε-caprolactone) (PCL) was recently explored by Xue et al. [61]. Modulating the hydrophilic–hydrophobic balance in the co-polymers allows for the modulation of the drug release and adaptable physical properties [61]. The researchers observed sustained anti-VEGF release, following encapsulation into the thermogel depots, in a relatively linear manner for up to 40 days in vitro [61]. Furthermore, the anti-angiogenic bioactivity of loaded anti-VEGF drugs was demonstrated using ex vivo choroidal implants from rats, as well as reduced vascular leakage in a neovascularization rabbit model. The co-polymerization of hydrophilic and hydrophobic components is typically observed in Pluronic triblocks as well, also known as poloxamers. Pluronic F-127 (i.e., poloxamer 407) has been widely explored for IVT ocular drug delivery [62,63,64,65]. The triblock consists of a central hydrophobic PPG flanked on either side by hydrophilic PEG blocks [62]. When combined with HA, two groups have recently observed positive results, with excellent injectability, abundant localization to the retinal tissue, good cell bioavailability, and controlled release with a superior anti-angiogenic effect when compared to ranibizumab suspension over 12 weeks [62,64]. They also showed that drug-in-liposome-in-hydrogel delivery was an option for those drugs that are not compliant with the hydrogel properties, such as flurbiprofen, a nonsteroidal anti-inflammatory drug (NSAID) for macular edema, thus expanding potential uses [63]. When combined with hydroxypropyl methylcellulose (HPMC), similar results were observed with the controlled delivery of bevacizumab-loaded nanoparticles for 256 h, in vitro degradation within 24 h, and therapeutic efficacy in rats with DR after 1 month [65]. However, there is a potential toxic effect following degradation by sonication which should be further explored prior to progressing to in vivo experiments [62].

Another approach for nanoparticle-associated drug delivery involves tragacanthic acid (TA) crosslinking with sodium acetate. The IVT administration of sunitinib-loaded nanoparticles in a TA-based hydrogel were shown to significantly halt DR pathophysiology through VEGF production regulation [90]. Polymer nanoparticle hydrogels have been explored in glaucoma and AMD as well [91]. Meany et al. observed that bimatoprost delivery formed depots in rabbit eyes, which degraded slowly over time, maintaining detectable drug levels in the vitreous humor for up to 8 weeks, while Tibbitt et al. observed that the delivery of small molecule drugs and model antibodies was successful in vivo with good tolerability [66,67]. Incorporating nanoparticles into the hydrogel improves the strength, stiffness, and degradation mechanics of the DDS in a concentration-dependent manner, which allows for finer tuning of the hydrogel for the drug and disease of interest [92]. However, in vivo toxicity after degradation is still a concern that must be considered [93].

Perhaps the greatest success in this field has been seen by Ocular Therapeutix (OTX), who have developed a PEG-based hydrogel called OTX-TKI (ClinicalTrials.gov ID: NCT 03630315) for the delivery of bevacizumab and the tyrosine kinase inhibitor (TKI), ataxinib. In a tolerability and pharmacokinetics study in Dutch belted rabbits, the group observed that significant levels of ataxinib could be observed in the vitreous humor, retina, and choroid for up to 6 months without clinically relevant toxicity, whereas there were low levels in the aqueous humor and plasma, pointing to a minimal risk of systemic toxicity [68,69]. The DDS was well tolerated, with no significant inflammatory response or safety concerns. In a 12-month trial, in which both ataxinib and bevacizumab were delivered, leakage was suppressed by 2 weeks and sustained for 12 months, whereas with bevacizumab monotherapy, the leakage returned after 4 weeks [70]. Drug concentrations remained high at all time points. Transitioning to cynomolgus monkeys as a non-human primate model, the DDS was again well tolerated with high levels for up to 6 months, especially in the retina and choroid/RPE layers [71]. Either three doses of 200 µg or one dose of 300 µg demonstrated the best results, with up to 50% release into the vitreous by 6 months [72]. Gel degradation occurred around 6 months, with released drug particles persisting in the vitreous for 3 months thereafter, regardless of the amount of drug loaded. Finally, in a phase one trial in patients who were either treatment-naïve or had a history of anti-VEGF therapy for neovascular AMD (nAMD), no ocular serious adverse events have been reported between 3 and 12 months, and the central subfield thickness had remained stable with subjects showing a clinically meaningful reduction in intraretinal and subretinal fluid by 2 months, maintained up to 13 months in one subject [73,74]. The implant was generally not visible after 9 months. A total of 80% of patients did not need rescue therapy for up to 7 months. OTX-TKI has proven to be a promising and effective therapy for nAMD and continues to undergo assessment in clinical trials towards eventual approval as a widespread alternative to current therapies, supporting sustained delivery and minimizing the need for repeated injections.

### 4.2. Natural Hydrogels for Intravitreal Ocular Drug Delivery

Other hydrogel materials are also beginning to be explored for IVT delivery, including HA and hyaluronic acid-methylcellulose (HAMC) and alginate–collagen gels. HAMC gel alone showed an increased survival of encapsulated regulatory T cells (Tregs), bridging IVT delivery with cell-based therapies [75]. There was a twofold increase in transferred Tregs in experimental autoimmune uveitis (EAU) mice, resulting in attenuated inflammation and the preservation of visual function compared to marginal therapeutic effects without hydrogel. When modified with SH3 binding peptides, there was a stable release of ciliary neurotrophic factor (CNTF) with successful delivery in mouse retina, leading to downregulated phototransduction genes in retinal degeneration [76]. Gels formed from HA are also being considered. Wang et al. found that aminated HA with beta cyclodextrin demonstrated long-term drug release for more than 60 days with a good cytocompatibility of ARPE-19 cells, another RPE cell line, as well as in vivo biocompatibility [77]. Further, after a single IVT injection in monkeys, Yu et al. observed that the delivery of bevacizumab in HA grafted with vinylsulfone showed a relatively constant drug concentration for at least 5 months in the eye, sufficient for the treatment of recurrent CNV compared to a bolus injection, which has a half-life of around 3.5 days [78]. These results demonstrate a significantly longer-lasting drug effect compared to that of the monthly IVT injections currently required, showing that hydrogel encapsulation effectively prolongs the therapeutic effect.

A composite–alginate–collagen hydrogel is also being explored by Wong et al., who found that the gel could be effectively terminated in vivo in rats through oral doxycycline to allow for further controlled therapy by avoiding surgical gel removal [79,80]. They observed therapeutic efficacy in rats with degenerating retina by reducing photoreceptor apoptosis and preventing retinal function loss. The combination of Dox termination technology with hydrogel DDS further demonstrates the flexible and modifiable nature of hydrogel therapies to better support the specific needs of the patient and disease. A supramolecular nanofiber hydrogel combined with calcium chloride salt (CaCl_2_) has also been explored recently for the delivery of betamethasone phosphate (BetP) and anti-VEGF for the treatment of AMD [81]. The hydrogel was fabricated by mixing BetP with the CaCl_2_, allowing for a simple formulation which minimizes in vivo exposure to many other molecules typically being tested, thus minimizing the risk of adverse events. The formulation enabled the sustained release of BetP to inhibit vascular proliferation in mouse retina, attenuate CNV, and scavenge ROS to reduce local inflammation. Antibody loading into the gel further showed sustained release for 14 days and decreased CNV lesions.

## 5. Suprachoroidal Drug Delivery Systems

The suprachoroidal injection represents an innovative technique for targeted drug delivery to the posterior segment of the eye. Utilizing the SCS, the region nestled between the sclera and the choroid, this method provides a promising avenue for minimally invasive medication administration [94]. Drug delivery into the SCS has several advantages over the previously discussed IVT injections for retinal disease. Due to the fibrous composition of the sclera, drug diffusion in the SCS faces lower resistance, thus drastically increasing the amount of medication that can reach the posterior eye segments compared to IVT and topical delivery [95,96]. Other benefits include achieving higher drug concentrations at the desired site, enhancing bioavailability, and extending the duration of the therapeutic effect [95,96]. Furthermore, this approach selectively compartmentalizes the treatment area, thereby reducing the likelihood of corticosteroid-related complications, such as the development of cataracts and the elevation of IOP [94,97,98]. Access to the SCS is typically achieved using microneedles, a minimally invasive procedure that allows for penetration through the sclera’s thickness without reaching the deeper choroid. This method enables the precise injection of drugs into the SCS, offering a targeted approach to drug delivery [94,97]. Integrating the superior targeting capabilities of suprachoroidal injections with the sustained-release potential of hydrogel encapsulation could present a highly effective therapeutic strategy for a range of ocular diseases.

Glaucoma, often referred to as the “silent thief of sight”, is a disease characterized by elevated IOP, which can lead to progressive damage to the optic nerve. This damage often results in visual field defects and, if left untreated, can cause irreversible blindness [99]. The disease may manifest with few or no symptoms in its early stages, making regular ophthalmic examinations crucial for early detection and treatment. Topical medication serves as the predominant therapeutic approach for managing elevated IOP and mitigating the progression of glaucomatous damage. However, this modality encounters challenges, including patient non-compliance, inconsistent IOP control, and the necessity for regular administration [100,101]. In contrast, drug-based hydrogel delivery to the SCS has emerged as a promising alternative. This innovative approach capitalizes on the augmented outflow of the aqueous humor through the alternative uveoscleral pathway, thereby offering a more rapid and sustained reduction in IOP [101]. Using an inert hydrogel microneedle injection into the SCS of cynomolgus monkeys and rabbits, it was shown that single and repeated injections significantly decreased IOP, with higher duration effects in rabbits. There was also a transient increase in eye wall thickness that lasted up to 3 months [102]. In fact, commercial HA-based hydrogels injected once or twice in the SCS kept low IOP values in rabbits for approximately 35 days before approaching its baseline values [101]. When comparing the suprachoroidal injection of a polyzwitterionic polycarboxybetaine (PCB) hydrogel, Hao et al. observed a reduction in IOP for at least 6 weeks [100]. Zwitterionic polymers can have dense hydration layers, giving it antifouling properties and preventing the adherence of unwanted molecules or contaminants to the surface [103,104]. They also have good biocompatibility and reduced cytotoxicity, excellent structural stability, and tunable regulation properties [103]. Hao et al. showed that their PCB hydrogel enabled longer IOP reduction compared to commercial HA gels [100]. The PCB gel had no inflammation or fibrosis, limited complications in a rabbit animal model, and a controllable gelation time [100]. However, Chae et al. demonstrated that with the correct modifications, a cross-linked and optimized HA hydrogel could reduce IOP in normotensive rabbits for up to 4 months [101]. Thiol-modified HA was crosslinked with PEG-DA. The cross-linked gel was better able to resist degradation and was well tolerated, with minor hemorrhage and fibrosis at injection sites. Comparatively, traditional therapies for reducing IOP are transient in nature and generally dissipate within hours [101].

In the management of rhegmatogenous retinal detachment (RRD), three primary surgical techniques are commonly employed: pneumatic retinopexy, scleral buckling, and primary vitrectomy, with or without adjunctive scleral buckling [105]. Scleral buckling closes retinal breaks through external scleral indentation, using carefully positioned buckling material to support the causative breaks [105]. However, this technique carries several complications, including increased IOP, necessitating fluid drainage or anterior chamber paracentesis, as well as induced myopia, anterior ocular ischemia, and diplopia, among others [105,106]. Considering these limitations, suprachoroidal hydrogel buckling has arisen as a promising alternative. Suprachoroidal hydrogel buckling has been explored by Szurman et al. using a cross-linked HA gel in both rabbits and in 21 patients with unilateral RRD [105,106,107]. In both cases, the gels were well tolerated, with a normal IOP and no visible retinal damage. The cross-linked gel showed visible buckling for approximately 8 weeks compared to around 1 week for a non-crosslinked gel. Retinal reattachment was observed in all but one patient at 8 weeks [107]. The formulated hydrogel is biocompatible, as HA is a significant native component of the eye and is also self-absorbable, allowing for safe biodegradability. This is also advantageous when retinal breaks are difficult to reach, circumventing the invasive nature of traditional buckling [106].

An HA-based hydrogel is also being explored for drug delivery in wet AMD [97,108,109]. The in situ forming HA hydrogel is cross-linked with PEG-DA and covers a large area following injection, extending to the posterior retina. The encapsulation of fluorescent polymer particles and later bevacizumab demonstrated successful delivery due to hydrogel swelling, pushing the gel to the retina [108,109]. Further, the gel could be tuned to decrease viscosity and mechanical strength, allowing for greater flow towards the retina, macula, and optic nerve. Bevacizumab release was observed for 6 months using this method, with good tolerance in rabbit eyes and slow-release as the gel degraded [108]. Finally, a recent study has explored the delivery of choroidal endothelial cells (CECs) using laminin-based hydrogels for AMD [110,111]. The gels demonstrated excellent cell survival in vitro and were well tolerated, with a minimal immune reaction in vivo in SGR rats [110,111]. There was widespread CEC survival and engraftment, suggesting that hydrogel-encapsulated cell delivery through suprachoroidal injection provides an alternative therapeutic avenue. Further work should continue to explore the long-term efficacy and, more importantly, safety profiles of suprachoroidal injections as well as optimizing the hydrogel composition for these various applications.

## 6. Cell-Based Therapies for Ocular Delivery

Recent research has increasingly concentrated on cell-based therapies, particularly in instances where vision loss or cellular degradation has reached an advanced stage, as seen in conditions like geographic atrophy in severe advanced AMD or end-stage retinitis pigmentosa (RP) with diffuse retinal atrophy involving the fovea. This focus on cellular replacement is compelling for several reasons. Not only does it offer the prospect of long-term benefits through the potential regeneration and restoration of native ocular functions, but it also possesses the distinct advantage of being mostly genotype-independent, unlike gene therapy approaches [112]. Much effort has been made in either the direct implantation of stem cells such as mesenchymal (MSCs), embryonic (ESCs) and induced pluripotent stem cells (iPSCs), or stem-cell-derived retinal and corneal cell types in various hydrogel formulations [113]. The following section discusses recent efforts in cell-based therapies for ocular disorders (Table 3).

### 6.1. Cell-Based Therapies for Diseases of Retinal Degeneration

Retinal degeneration constitutes a diverse array of disorders characterized by the loss of photoreceptors, which inevitably leads to vision impairment. This category includes, but is not limited to, conditions such as RP, AMD with geographic atrophy, and hereditary retinal dystrophies [113]. Specifically, AMD stands as one of the leading causes of vision loss globally, yet the available treatment options are often limited in scope and efficacy [113]. HA is a prominent component of the eye and thus has been explored as a hydrogel-delivery system for cell-based therapies. Wang et al. observed that HA hydrogels as a 3D bioprinting scaffold successfully allowed for the differentiation of retinal progenitor cells (RPCs) into photoreceptors in vitro with the support of RPEs cells [114]. Likewise, Mitrousis et al. observed successful vision recovery in mice who were completely blind upon the co-transplantation of RPE and photoreceptor cells in an HAMC hydrogel [115]. Co-transplantation was superior to the transplantation of either cell type alone, given that it better supports the complex native retinal structure [115]. HA hydrogels have been widely used and studied due to their biocompatibility, natural presence, biodegradability, and versatility [146]. However pure HA does not form a suitable gel on its own, thus complicating its use [115].

The co-polymerization of HA with gellan gum has also been explored recently, as gellan gum is a widely used additive with a high water retention capacity and acts as a gelling agent, allowing for cell proliferation [147]. Youn et al. showed that moderate amounts of HA improved the gel properties for retinal injection and allowed for cell encapsulation and gelation, cell compatibility and proliferation, as well RPE gene expression in vitro [116]. Co-polymers allow for some flexibility in modifying the hydrogel composition to best suit the needs of the cellular cargo for survival and proliferation. Gellan gum-based co-polymer hydrogels have been studied in combination with PEG, dopamine, gelatin, and chitosan as well [117,118,119]. The results generally demonstrate that altering the gel composition allows for better suitability for RPE regeneration and delivery and affects gel characteristics—i.e., the pore size, gelation time, and degradation time, all of which affect cell encapsulation and successful proliferation [117,118,119]. All combinations demonstrated successful RPE cell proliferation and mature gene expression with the increased secretion of ECM components necessary to maintain native retinal integrity. However, these trials demonstrate in vitro success with a need to focus on the translation to in vivo long-term studies.

Gelatin-based hydrogels have been the recent focus for in vivo retinal applications. Gelatin-hydroxyphenyl propionic acid (HPA) gels have been studied as cell carriers for human RPCs, demonstrating good cell viability compared to that of conventional fibronectin and 2D scaffolds, a faster gelation time, good proliferation, and a more widespread distribution of encapsulated cells in the subretinal space whilst limiting apoptotic and immune responses when compared with respective controls [121,122,123]. An important consideration when working with gelatin-HPA gels, however, is minimizing the effects of hydrogen peroxide—the gel cross-linker—on oxidative stress in vivo [122]. The short-term results thus far demonstrate that the gels are protective for cells that may experience high stress during encapsulation, an important consideration for posterior eye delivery to reach structures such as the retina [123]. The addition of polydopamine has also been studied in gelatin hydrogels by Tang et al., who demonstrated similar results showing improved cell adhesion, proliferation, and differentiation into retinal neuronal cells in the presence of dopamine when compared to those without [124]. Dopamine serves as a protective factor for encapsulated cells and is responsible for enhancing neurite outgrowth and neuronal development [124,148].

Likewise, alginate-based hydrogels have also been a recent focus, where alginate hydrogel alone demonstrated superiority when compared to gelatin with regard to the viability and proliferation of MSCs [128]. The addition of beta-carotene further elevated the number of differentiated cells [128]. The presence of curcumin in the alginate hydrogels also enhanced cell proliferation and retinal differentiation in vitro [129]. Alginate gels with the addition of the arginine–guanidine–aspartate (RGD) peptide have also been studied. The RGD peptide is well known for promoting cell attachment to a variety of biomaterials [130]. Here, its combination with alginate proved to effectively enhance the generation and survival of RPE and optic vesicles, the RPE formation from stem cell lines, and the formation of a neural retina after more than 30 days in vitro and 1 week in vivo [130,131]. The gel also demonstrated superiority compared to gelatin–HA gels [130].

Another common consideration for retinal hydrogels is fibrin—a prime candidate given its natural role as a coagulating factor. Fibrin gel is a strong candidate for a 3D scaffold, allowing for cell differentiation into photoreceptor-like cells, with a demonstrated superiority in cell adhesion, viability, and photoreceptor markers, as well as the secretion of growth factors and in vivo cell survival [132,133,134]. Given that fibrin is naturally occurring, it does present the risk of rapid degradation by the tissue plasminogen activator, which may become problematic for cell differentiation and engraftment in the long term as the gel is degraded [134]. Regardless, when compared with gelatin methacryloyl (GelMA), HA, and alginate gels, fibrin demonstrated excellent cell survival and superiority among all the groups [135]. This should, however, be further explored, especially given that all of these gels have demonstrated success in other studies. Cell encapsulation in a GelMA composite with chitosan microspheres successfully resulted in well-spread and viable cells in a recent preliminary in vitro study [136]. Chitosan has a poor aqueous solubility despite its beneficial effects on cell delivery, and encapsulation in the GelMA allowed for the use of chitosan in highly aqueous environments such as the eye [136]. The varying levels of success in these studies do point out the nuances of hydrogel formation and the importance of the hydrogel composition when considering long-term cell survival and delivery. Chitosan has also been explored as a hydrogel component in a chitosan hydrochloride-oxidized dextran gel, which serves to combat the poor solubility, allowing for uniform cell delivery and favored RPC proliferation and differentiation into neurons in vitro [137]. Other hydrogels currently under exploration for cellular delivery such as NIPAAm and polymers like PEG-poly(L-lysine)-poly(allylamine) (PEG-PLL-PAA) and PCL have demonstrated initial success in supporting various stem cells for retinal regeneration, with significant exploration still required [110,149,150,151].

### 6.2. Cell-Based Therapies for Corneal Damage

Corneal damage can occur due to a range of etiological factors, including but not limited to bacterial or viral infections, autoimmune-mediated inflammation, mechanical trauma, chemical burns, and corneal dystrophies. These etiologies can lead to various pathological changes such as stromal scarring, endothelial cell dysfunction, or the depletion of limbal stem cells, each of which has the potential to significantly compromise vision [152,153]. Current treatment modalities for such advanced corneal damage include options like penetrating keratoplasty (PKP), Descemet’s membrane endothelial keratoplasty (DMEK), and Descemet’s Stripping Automated Endothelial Keratoplasty (DSAEK). While these treatments can be moderately effective in restoring or improving vision, they rely on allogeneic corneal grafts [153]. However, this approach faces a critical challenge: a significant global shortfall in available donor corneas, which currently meets only 1.4% of the existing demand. With the incidence of corneal injuries on an upward trajectory, there is an urgent need for alternative corneal graft sources [153].

Silk fibrin hydrogels were used to successfully encapsulate human corneal stromal cells (HCSCs), with up to 95% light transmittance, homogenous morphology, and greater cell adhesion and proliferation compared to controls [138]. They also resulted in longer gelation times and improved cell viability [138]. Peptide-based hydrogels have also been widely studied for corneal regeneration due to their innate biocompatibility and degradation and their ability to mimic native ECM. Both di-propargylated peptides with PEG tetraazide and poly-ε-lysine hydrogels demonstrated a good adherence and attachment of HCECs while maintaining transparency [139,140,141]. The functionalization of the hydrogel with RGD peptides further increased cell adhesion and expansion, with a uniform monolayer forming at 5 weeks in vitro [139].

The co-administration of cell-based therapies with existing medications in hydrogels provides an appealing avenue for maximizing treatment efficacy and targeting multiple systems with a singular administration. HCECs embedded in an HA-beta cyclodextrin hydrogel with the co-encapsulation of DEX were well supported, remained stable, and aided in the sustained release of DEX for 5 days in vitro [142].

Hydrogels, which have been extensively studied for in situ tissue engineering, provide an established avenue onto which ocular cell therapies have also been explored in recent years. These include collagen-based hydrogels—an appealing material given its widespread presence in human connective tissue and structural function [143]. However, collagen is a protein and requires modifications to be stable enough for use as a hydrogel scaffold [144]. Modifications with PEG, fibronectin, and gelatin cross-linked with 1-ethyl-3-(3-dimethylaminopropyl)carbodiimide) (EDC) and N-hydroxysuccinimide (NHS) resulted in transparent gel formations with an improved tensile strength and elastic modulus comparable to the native retina, a good secretome of encapsulated cells for in vitro and in vivo biocompatibility for up to 1 week, and modulated cell spreading and proliferation [143,144,145].

Gelatin-based hydrogels have also been explored with other modifications for ocular applications. A gelatin–chitosan gel with loaded iPSC-MSC-derived exosomes promoted corneal repair in rats for up to 1 week and suppressed translocation-associated membrane protein 2 (TRAM2), a modulator of collagen biosynthesis, to avert excessive ECM deposition [125]. Gelatin glycidyl methacrylate (GM), a modification of GelMA, when delivered alongside N-vinylpyrrolidone (VP) at optimal ratios, resulted in a high tensile strength and well-rounded properties suitable for cell encapsulation for corneal blindness [126]. The presence of a synthetic component allowed for stability against collagenase while maintaining cell adhesion, proliferation, and migration in vitro. However, the mechanical properties of the gel were weaker than those of native corneas, prompting in vivo studies to observe how it would hold out long-term. A GelMA + polyethylene glycol diacrylate gel was also formed, and again, optimal ratios allowed for reasonable corneal stromal cell viability and gene expression as well as gel strength [127]. The incorporation of collagen nanofibers into the gel further improved biomimetic properties, demonstrating the versatility and benefits of naturally derived materials in hydrogels. A methacrylate hydrogel formed with gellan gum and cross-linked with lithium phenyl-2,4,6 trimethylbenzoylphosphinate (LAP) enhanced corneal endothelial cell encapsulation and improved the compressive strength of the gel compared to GM alone [120]. However, the cross-linked gel had a lower transparency, highlighting the challenges of balancing gel integrity with biological efficacy. Various hydrogels have thus shown initial efficacy in treating corneal damage while maintaining eye transparency. Future work should continue to explore the in vivo and long-term efficacy and biodegradability of the embedded hydrogels.

### 6.3. Limitations of Hydrogel in Cell-Based Therapies for Retinal Diseases

While hydrogels are showing initial promise in allowing for safe cell delivery to diseased retina and the successful engraftment of transplanted cells for eventual self-sustaining regeneration, proof-of-concept studies are severely lacking [13,154,155]. Most studies are currently in ex vivo stages, and those progressing to in vivo are primarily demonstrating short-term effects—typically 1–2 weeks. Another concern is the lack of coherence when it comes to hydrogel compositions and the types of cells delivered, serving to hinder significant progress beyond initial stages as each formulation requires a rigorous process of evaluating optimal concentrations, biocompatibility, cytotoxicity, and cell support. Focusing advancements on a few promising candidates may help to progress the development of successful hydrogel scaffolds more rapidly so they are ready for clinical use.

## 7. Utilization of Hydrogels in Intraocular Lens Technology

In cataract surgery, the eye’s natural lens, which has become clouded, is surgically removed and replaced with an artificial intraocular lens (IOL). Traditionally, IOLs have been fabricated from a variety of materials including poly(methyl methacrylate) (PMMA), hydrophilic acrylate, hydrophobic acrylate, silicone, and collamer. PMMA was initially favored for its biocompatibility and optical clarity [156]. However, advancements in material science have led to a shift towards more foldable materials, such as acrylic polymers, which permit IOL insertion through smaller incisions, thereby improving surgical outcomes [156].

Incorporating hydrogels into acrylic intraocular lenses (IOLs) has shown promise in reducing the incidence of posterior capsular opacification (PCO), a common complication following cataract surgery [157]. PCO is caused by the adhesion and proliferation of residual lens epithelial cells, and surface modification is a key approach to mitigating this issue. Specifically, hydrogels like poly(ethylene oxide) (PEO) have been effective in minimizing protein deposition and cell adhesion on the IOL surface [157]. A study by Lin et al. further substantiated the benefits of hydrogels by showing that PEG, a semi-IPN hydrogel brush coating, can enhance the biocompatibility of IOLs when applied through surface-initiated reversible addition-fragmentation chain transfer (SI-RAFT) polymerization, ultimately reducing the occurrence of PCO in vivo [158]. While the management of posterior capsular opacification (PCO) post-cataract surgery is relatively straightforward, involving a YAG laser capsulotomy as an in-office procedure with predictable outcomes, there may be advantages to using IOLs that lower the risk of PCO development. These benefits could include not only a reduced likelihood of complications such as IOL movement, refractive changes, IOL surface damage, iritis, uveitis, elevated IOP, cystoid macular edema (CME), and retinal detachment but also cost-saving implications by avoiding additional ophthalmologist visits and procedural fees [159].

Incorporating pharmaceutical agents into IOLs offers a compelling avenue for enhancing postoperative outcomes, thanks to the higher bioavailability these drug-loaded IOLs can provide when compared to conventional ophthalmic drops. The latter are often subject to variable individual usage and reduced tissue bioavailability due to lacrimal drainage. In addition to enhancing bioavailability, IOLs can sustain prolonged drug release, mitigating the need for frequent topical applications. Several studies have explored this concept. For instance, Li et al. investigated hydrogel IOLs enriched with β-cyclodextrin (β-CD), revealing that this modification not only improves the IOL’s mechanical properties but also enables sustained DEX release post-cataract surgery [160]. These drug-delivering hydrogel-based IOLs could streamline postoperative care by obviating the need for frequent topical corticosteroids, traditionally applied four times daily for 2–4 weeks to counter post-operative anterior chamber inflammation. Additionally, these DEX-releasing IOLs could serve as a prophylactic measure against pseudophakic CME [161]. Nevertheless, the incorporation of drugs like DEX into IOLs is not without drawbacks. Artigas et al. have shown that pHEMA-silicone IOLs loaded with DEX can compromise the lens’s optical properties [162]. Furthermore, a pertinent clinical consideration arises for patients known as steroid responders, who exhibit an elevated IOP when exposed to steroids. While topical steroid administration permits rapid discontinuation in such cases, a DEX-releasing IOL would present a more complex predicament. Ceasing drug release would necessitate IOL explantation, a procedure fraught with added complexities and comorbidities. Thus, the pharmacological customization of hydrogel-based IOLs warrants careful patient selection and underscores the need for additional safety studies.

Beyond their utility in IOL-based drug delivery, hydrogels are also being explored for their potential as intraocular disease sensors. For instance, Shin et al. have innovatively utilized diacrylamide-group-modified poly(ethyleneglycol) (PEGDAAm) hydrogels to fabricate a fluorogenic intraocular lens (FIOL). This FIOL demonstrates in vivo reactivity to metalloproteinases (MMPs) in the aqueous humor, thereby acting as a potential early-stage biomarker for Alzheimer’s disease [163].

## 8. Conclusions

The dynamic landscape of ophthalmology is witnessing transformative shifts, with innovations poised to reshape therapeutic and diagnostic modalities. Hydrogels, given their inherent characteristics, have emerged as a versatile and promising material in addressing multifaceted challenges in ocular care. This review has provided a comprehensive exploration of hydrogel applications, spanning from optimizing DDS for the posterior segment to refining intraocular lens technology.

The anatomical intricacy of the eye, especially the posterior segment, requires tailored therapeutic approaches, which hydrogels adeptly address. Their potential to ensure controlled and sustained drug release in areas like the retina and choroid illuminates their potential in minimizing invasive interventions, enhancing patient comfort and compliance, as well as improving overall treatment outcomes. Similarly, in intraocular lens technology, hydrogels are being employed not only to enhance biocompatibility and reduce complications but also to integrate advanced features such as post-operative drug delivery and disease sensing.

Yet, like all innovations in medical science, the adoption of hydrogel-based interventions necessitates rigorous evaluation. The safety, efficacy, and long-term implications are paramount considerations. As of the current time, most studies are at the preclinical stage, highlighting the necessity for further comprehensive clinical trials and investigations. While hydrogels hold immense promise, patient-specific factors, potential complications, and the broader clinical context, along with the nascent stage of many of these studies, should guide their utilization. Efforts must be intensified to transition these promising preclinical findings into tangible clinical applications, ensuring their safety and efficacy in real-world settings.

In summary, the marriage of hydrogels with ocular therapeutics epitomizes the synthesis of material science and medicine, ushering in a new era of patient-centric care. With ongoing research, it is anticipated that hydrogels will solidify their place in the ophthalmologist’s armamentarium, bridging the gaps in current treatment paradigms and paving the way for more effective and personalized patient care.

## Figures and Tables

**Table 1:** Categories and types of polymers involved in hydrogel synthesis.

Examples of Types of Polymers	Advantages	Disadvantages	References
Natural source polymers for hydrogels			
Collagen, chitosan, gelatin, alginate, hyaluronic acid	Biodegradable Non-toxic Originate from renewable sources Easily interact with biological components Easy penetration of encapsulated cells into the region of interest	Low mechanical resistance and strength Rapid degradation Weak stability Shorter polymer chains Require modifications	[7,8,20,21]
Synthetic source polymers for hydrogels			
Poly(ethylene glycol) (PEG), poly(acrylic acid) (PAA), N–isopropyl acrylamide	Greater water absorption Expanded library of materials Improved mechanical strength and stability Greater control of gel composition and mechanical properties Longer half-life allowing for sustained delivery	Lower cytocompatibility Greater foreign body response May not be a suitable environment for cell adhesion without additional modifications More complex and thus riskier to use	[9,20,21,26]
Semi-synthetic source polymers for hydrogels: synthetic polymers conjugated to ECM ^a^ components			
PEG + albumin, PEG + gelatin, PEG + fibronectin	Combined benefit of natural and synthetic polymers Increased mechanical strength and stability Good biocompatibility Controlled degradation rate Good cell adhesion, survival, growth, and proliferation Controlled use of specific ECM components provides a range of strength to the gel	Increased complexity of gels presents a challenge with cytocompatibility Currently underdeveloped	[21,27]

^a^ Abbreviations: ECM: extracellular matrix.

**Table 2:** Summary of recently studied ^a^ hydrogels for the treatment of ocular diseases by intravitreal delivery.

Material Type ^b^	Indication ^b^	Drug Delivered	Key Features	Challenges and Considerations	Current Usage	References
NIPAAm-PEG-NIPAAm triblock polymers	PDR; DME; RVO	DEX	Self-healing Thermosensitive copolymer Increased DEX residence time Long-lasting drug release	No in vivo analysis to assess safety and biocompatibility	Pre-clinical: in vitro	[51]
PEG + NIPAAm-based hydrogel composites	Glaucoma; wAMD with CNV; PDR; DME; RVO	Vancomycin, aflibercept, ranibizumab	Long-term in vivo sustained release Biocompatibility Tested in non-human primate models Body temperature-responsive	Further testing required in vivo for diseased models Not always biodegradable	Pre-clinical: in vitro (computational models) and in vivo	[52,53,54,55,56,57]
mPEG-PLGA-BOX	wAMD	Bevacizumab	sol-gel phase transition in body temperature Improved biocompatability and biodegradation	Extended release was not assessed	Pre-clinical: in vitro and in vivo	[58]
PLGA-PEG-PLGA triblock copolymer	Neurodegeneration of the retina	DEX	Ease of co-delivery of drugs’ micelle-like structures with a high hydrophobic core Thermoresponsive hydrogel	Drug properties are essential to consider for co-delivery (e.g., hydrophobicity)	Pre-clinical: in vitro	[59]
PBLA-PEG-PBLA triblock copolymer	Retinitis caused by cytomegalovirus	Ganciclovir	Majority of drug release within 96 h In situ formation Thermosensitive	In vivo testing with IVT delivery required	Pre-clinical: In vitro	[60]
Poly (PEG/PPG/PCL) urethane	wAMD; PDR	Bevacizumab, aflibercept	Thermosensitive Adjustable hydrophilic–hydrophobic balance to modulate the release rate (tunable release) Non-toxic to endothelial cells In vivo efficacy	More in vivo testing required to characterize the functional benefit	Pre-clinical: in vitro and in vivo	[61]
Poloxamer-based hydrogels	Non-specific neovascularization	Ranibizumab, flurbiprofen, bevacizumab	Hydrogel-based NP delivery for a specialized effect on target cells NPs encapsulate molecules sensitive to in vivo degradation Biodegradable Hydrogel/nanoparticle co-loading extends the release time	Unclear of the extended time of release in vivo Specialized retinal targeting is difficult Drug loading may accelerate the gelation time	Pre-clinical: in vitro and in vivo	[62,63,64,65]
Polymer nanoparticle hydrogels	Glaucoma	Bimatoprost	Shear thinning Self-healing Slow release of molecular cargo in the vitreous humor	Mild foreign body response	Pre-clinical: in vitro and in vivo	[66,67]
PEG-based OTX-TKI hydrogels	wAMD	Bevacizumab, ataxinib	Biodegradable 12-month sustained efficacy after a single dose Success in non-human primates Injectable at room temperature Viscoelastic Sol-gel transition at body temperature	In vivo efficacy should be further explored Mild foreign body response	Pre-clinical: in vitro and in vivo Clinical Phase I trial	[68,69,70,71,72,73,74]
HAMC-based hydrogels	wAMD; RP	Tregs, CNTF	Good vitreous humor substitute material, so safe for the eye SH3 to control the release of CNTF In vivo bioactivity	Unclear how long the drug is bioactive beyond 7 days Long-term studies required + functional studies	Pre-clinical: in vivo	[75,76]
HA-based hydrogels	wAMD; endophthalmitis	Bevacizumab, voriconazole	Swelling resistance Sustained drug release Transparent	Further studies required to assess how long treatment can be maintained In vivo studies required Unclear safety risks	Pre-clinical: in vitro and in vivo	[77,78]
Composite alginate–collagen gels	wAMD; RP	GDNF as a sample delivery molecule	Long-lasting drug delivery Functional improvement in vivo	Unclear biodegradability Consistent topical application of antibiotics following injection Challenge of encapsulation when using proliferative cells	Pre-clinical: in vitro and in vivo	[79,80]
Drug-loaded nanofiber hydrogel + CaCl_2_	wAMD	BetP	Dual action as anti-VEGF and anti-inflammatory No additional molecule required A previous study showed that an antibody can be loaded into this gel as well Shear-thinning and self-healing	Limits the drugs that can be used to form this gel system and that successfully gel with CaCl2	Pre-clinical: in vitro and in vivo	[81]

^a^ Regroups recent studies from 2017 to the present; ^b^ Abbreviations: AMD: age-related macular degeneration (wAMD: wet AMD), BetP: betamethasone phosphate, CNTF: ciliary neurotrophic factor, CNV: choroidal neovascularization, DEX: dexamethasone, DME: diabetic macular edema, DR: diabetic retinopathy, GDNF: glial cell line-derived neurotrophic factor, HA: hyaluronic acid, HAMC: hyaluronic acid methylcellulose, IVT: intravitreal, ME: macular edema, NIPAAm: N-isopropylacrylamide, NP: nanoparticle, OTX: Ocular Therapeutix, PBLA: poly(β-benzyl l-aspartate), PCL: polycaprolactone, PDR: proliferative diabetic retinopathy, PEG: poly(ethylene glycol), PLGA: poly(lactic-co-glycolic acid), PPG: poly(propylene glycol), RP: retinitis pigmentosa, RVO: retinal vein occlusion, TKI: tyrosine kinase inhibitor, Tregs: regulatory T cells, VEGF: vascular endothelial growth factor.

**Table 3:** Summary of recently studied ^a^ hydrogels for ophthalmology-oriented cell-based treatments.

Material Type ^b^	Indication	Cell Type/Drug	Key Features ^b^	Challenges and Considerations	Current Usage	References
HA-based 3D bioprinting	Retinal cell regeneration	RPCs	HA gels can be functionalized to match the physical and chemical properties of native retina	Ensuring successful injection and integration into the retina	Pre-clinical: in vitro	[114]
HAMC hydrogel	Retinal degenerative diseases	RPEs + photoreceptor cells	Support the survival and proliferation of RSPC in vitro and their injection into retina	Ensuring successful injection and integration into the retina	Pre-clinical: in vitro and in vivo	[115]
Gellan gum-based hydrogels	RPE cell regeneration for RP, AMD, and hereditary retinal dystrophies	RPEs	Tunable properties to suit the disease and cell of interest Thermo-responsive gelation Good biocompatibility Mimics ECM quite well and allows for well-distributed cell attachment	Gellan gum has a high molecular weight Gellan gum is a weak scaffold on its own, so must be combined with a different biomaterial, complicating the chemistry	Pre-clinical: in vitro	[116,117,118,119,120]
Gelatin-based hydrogels	Cell replacement for retinal diseases, corneal damage	RPCs, RPEs, MSCs, CSCs	In situ cross-linking Mimics ECM and can alter components based on need Enhances cell proliferation Suitable 3D cell scaffold Good transparency	Need more in vivo studies to characterize biocompatibility and successful injectability	Pre-clinical: in vitro and in vivo	[121,122,123,124,125,126,127]
Alginate hydrogels	Retinal degenerative diseases	MSCs, RPEs	Differentiation of MSCs on a 3D scaffold Addition of RDG enhances cell adhesion Biocompatibility of natural polymers	No in vivo testing of the 3D scaffold	Pre-clinical: in vitro	[128,129,130,131]
Fibrin-based hydrogels	AMD and retinal degeneration	MSCs, RPEs	Tunable properties by varying individual concentrations Successful use in many other systems and proven safety in many animal models Stem cell protection during delivery Superiority in retinal cell transplantation compared to gelatin- or HA-based gels	Rapid degradation by a tissue plasminogen activator Opaque gel presenting issues with vision Challenge with the surgically induced maintenance of the monolayer Ex vivo cell differentiation on a scaffold may not succeed in vivo	Pre-clinical: in vitro and in vivo	[132,133,134,135]
GelMA hydrogel + chitosan microspheres	AMD	RPEs	Hydrogels can provide protection for a 3D cell culture Mimics the ECM Hydrogel aggregates cell microspheres to control cell spread and dispersion Complete rapid degradation	Need for in vivo studies in animal models for safety and long-term efficacy	Pre-clinical: in vitro	[136]
Chitosan hydrochloride with oxidized dextran	RPE cell regeneration for AMD and retinal degeneration	RPEs	Dextran facilitates cell proliferation Gelation time can be manipulated by varying the concentration of chitosan Self-healing	Ensuring successful injection and integration into the retina Need long-term data for the success of ocular transplantations Fibrin gels require anti-fibrinolytic agents to control the degradation profile	Pre-clinical: in vitro and in vivo	[137]
SF and PAA hydrogel	Corneal stromal tissue regeneration	CSCs	Facilitated keratocyte migration during healing, improved cellular adhesion, and no cytotoxicity SF helps to overcome the brittle nature of pure PAA hydrogels In situ formation of the gel limits the risk of infection and strengthens the interaction between the tissue and gel	Gel still demonstrated brittle mechanical properties Corneal stroma is difficult to engineer due to the need for transparency	Pre-clinical: in vitro	[138]
Peptide-based hydrogels	Cell replacement for corneal and retinal diseases	HCECs	Peptide inclusion enhances cell adhesion and proliferation Versatile and can be manufactured to mimic ECM Good transparency	Corneal endothelial cells have a limited replicative capacity High porosity and 3D structure may not be best suited for the formation of epithelial/endothelial cell monolayers	Pre-clinical: in vitro and in vivo	[139,140,141]
HA and beta-cyclodextrin hydrogel	Non-specified corneal damage	HCECs + DEX	Combined cell and drug delivery for enhanced therapy	Quantity and homogeneity of drug loading in a hydrogel system may be limited	Pre-clinical: in vitro	[142]
Collagen-based modified hydrogels	Corneal alkali burns	MSCs, LESCs	Superior release of secreted factors onto an ocular surface Combination gels enhance overall mechanical properties and degradation	Wound healing similar to topically applied MSCs calls into question the need for surgical intervention Pure collagen hydrogels are weak and degrade rapidly due to the high water content	Pre-clinical: in vitro	[143,144,145]

^a^ Regroups recent studies from 2017 to the present; ^b^ Abbreviations: AMD: age-related macular degeneration, CSC: corneal stromal cells, DEX: dexamethasone, ECM: extra-cellular matrix, GelMA: gelatin methacryloyl, HA: hyaluronic acid, HAMC: hyaluronic acid methylcellulose, HCEC: human corneal epithelial cells, LESC: limbal epithelial stem cells, MSC: mesenchymal stem cells, PAA: poly(acrylamide), RGD: arginine-glycine-aspartate peptide sequence, RP: retinitis pigmentosa, RPE: retinal pigment epithelium, RPC: retinal progenitor cells, SF: silk fibrin.

## Data Availability

Not applicable.
